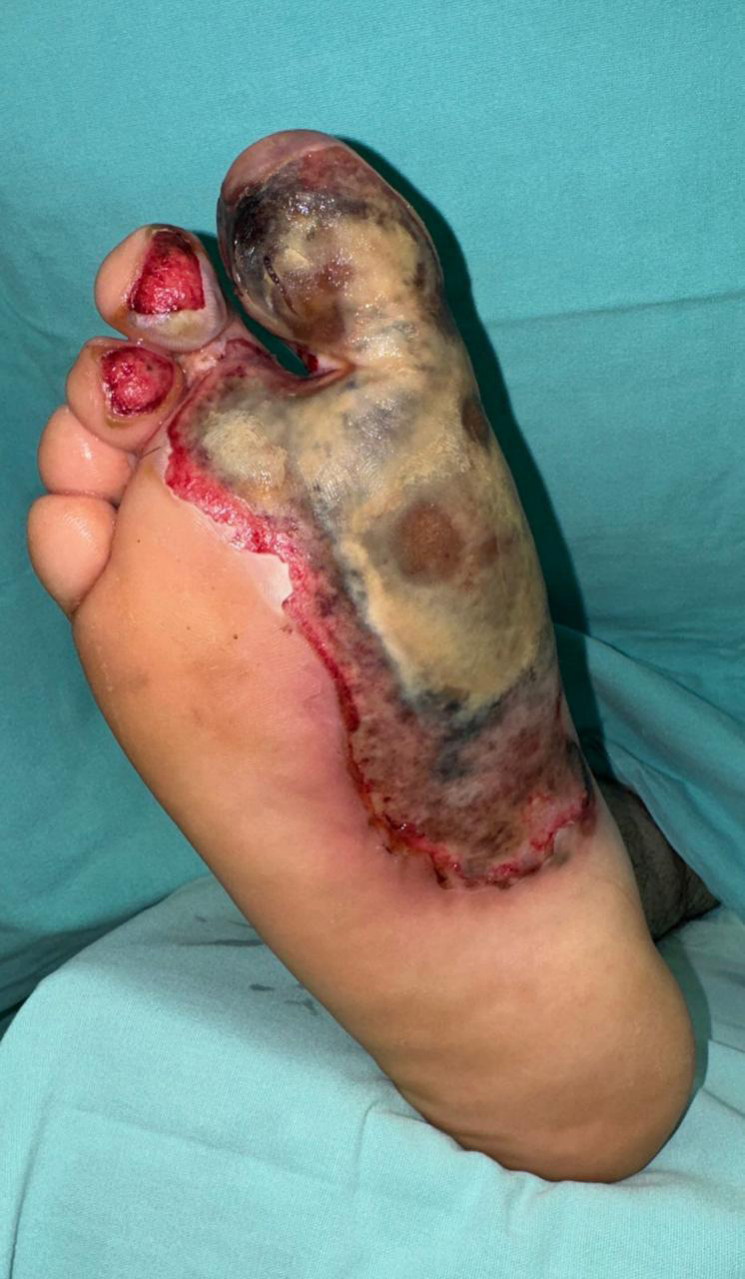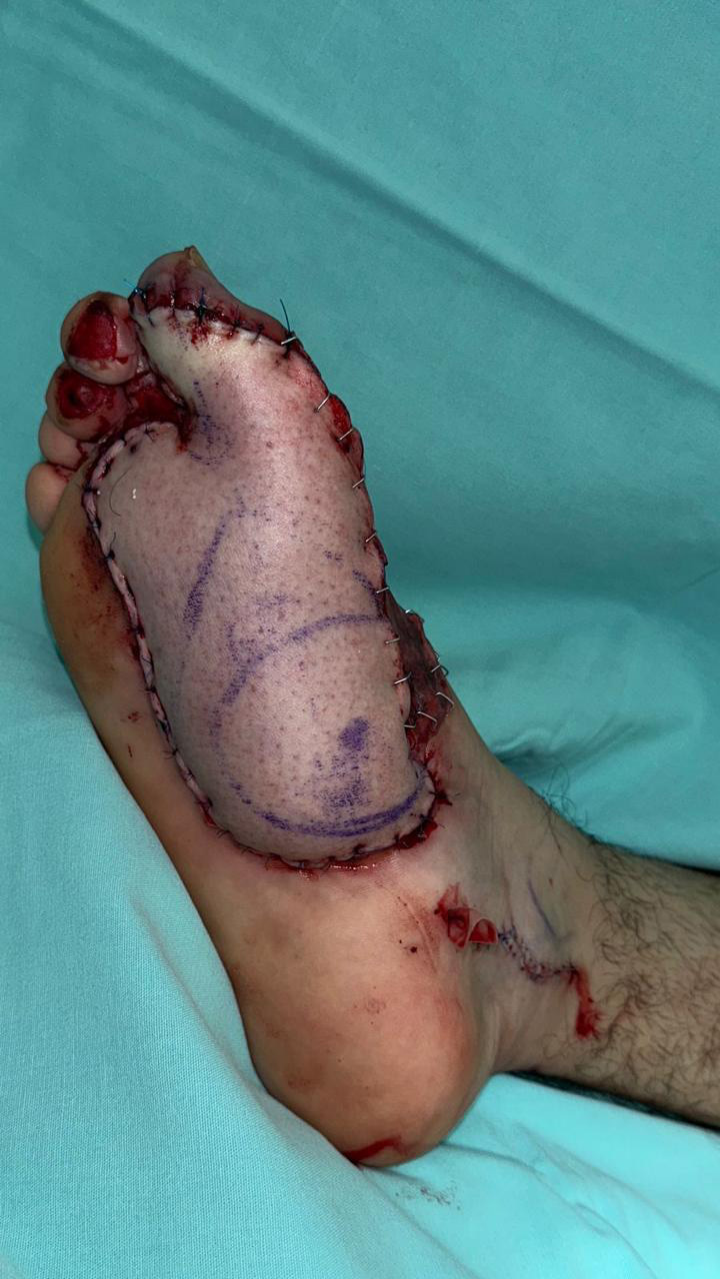# 847 Plantar Reconstruction with Flap for Sequelae of Electrical Burn: A Case Report and Surgical Management

**DOI:** 10.1093/jbcr/iraf019.378

**Published:** 2025-04-01

**Authors:** Bernardo Fernández Reyes, Lucia Cano Pérez, Cesar Alejandro González Martinez, Alejandro Flores Uribe, Adrian Gonzalez Martinez

**Affiliations:** Christus Muguerza Alta Especialidad; Christus Muguerza Alta Especialidad; Hospital Universitario Dr. José Eleuterio González; Hospital Universitario Dr. José Eleuterio González; Christus Muguerza Alta Especialidad

## Abstract

**Introduction:**

Electrical injuries occurs when energy current travels through the body due to contact with an electrical source. The direct effect of electrical current and the conversion of electrical to thermal energy, can cause tissue damage and organ dysfunction. We present a case of a 23-year-old male who came to the emergency department with a high voltage electrical injury in face and feet. Second and third degree burn on face, and third degree burn on soles of both feet and right foot extension to dorsum, a total of 4% total body surface area.

**Methods:**

Washout and debridement were performed under sedation and covered with nitrofural on feet and an ointment with bacitracin, neomycin and Polymyxin B was applied to the face. Subsequently the patient underwent surgery, first debridement, irrigation and washout were performed on the right foot, removing neurotic tissue. The plantar fascia and the first metatarsal were identified, once we had the defect without necrotic tissue, a flap was designed according to the resulting defect. An incision was made on the medial side of the right foot, identifying the posterior tibial artery and two veins. In the left thigh, perforators of the anterolateral thigh (ALT) flap were identified. The flap was dissected from medial to lateral, identifying the musculocutaneous perforator, which was continued to the lateral femoral circumflex artery, with a pedicle of approximately 10 cm. Likewise, when lifting the flap, a sensory branch of the cutaneous nerve was identified, which was preserved along with the flap. Then the flap was released and taken to the defect by performing anastomosis, two end-to-end veins and the end-to-side artery. The interdigital nerve was identified in the right foot, in which nerve anastomosis was performed with the sensory nerve of the flap. Then a graft was taken and applied from the back of the same foot.A debridement and washout were performed and a graft was applied from the plantar area of the left foot. Then an ointment with bacitracin, neomycin and Polymyxin B was applied.

**Results:**

Patient follow-up was per hour with the evaluation of the flap throughout 5 days. After this, the patient was discharged without complications related to the flap. The patient was followed weekly with adequate capillary refill, coloring and without sign of ischemia

**Conclusions:**

Anterolateral thigh flap is a useful strategy for reconstruction of vessel, nerves and soft tissue in electrical injuries of damaged lower limbs.

**Applicability of Research to Practice:**

Although this strategy has a higher risk of failure than with other causes of burn, the results shows that this technique is useful and feasible.

**Funding for the Study:**

N/A